# Lymph node ratio and hematological parameters predict relapse-free survival in patients with high grade rectal neuroendocrine neoplasms after radical resection: a multicenter prognostic study

**DOI:** 10.1186/s12957-023-03144-0

**Published:** 2023-09-22

**Authors:** Xinyu Zeng, Peng Zhang, Guangsheng Zhu, Chengguo Li, Rui Zhang, Minhao Yu, Guole Lin, Maojun Di, Congqing Jiang, Yong Li, Yueming Sun, Lijian Xia, Pan Chi, Kaixiong Tao

**Affiliations:** 1 Department of Gastrointestinal Surgery, Union Hospital, Tongji Medical College, Huazhong University of Science and Technology, Wuhan, 430022 China; 2 Department of Gastrointestinal Surgery, Hubei Cancer Hospital, Tongji Medical College, University of Science and Technology Huazhong, Wuhan, China; 3 Department of Colorectal Cancer, Liaoning Cancer Hospital & Institute, Shenyang, China; 4 Department of Gastrointestinal Surgery, Renji Hospital, School of Medicine, Shanghai Jiao Tong University, Shanghai, China; 5 Department of General Surgery, Peking Union Medical College Hospital, Chinese Academy of Medical Sciences, Beijing, China; 6 Department of Gastrointestinal Surgery, Taihe Hospital, Hubei University of Medicine, Shiyan, China; 7 Department of Colorectal and Anal Surgery, Zhongnan Hospital of Wuhan University, Wuhan, China; 8 Department of Gastrointestinal Surgery, Department of General Surgery, Guangdong Provincial People’s Hospital (Guangdong Academy of Medical Sciences), Southern Medical University, Guangzhou, China; 9 Department of Colorectal Surgery, Jiangsu Province Hospital, Nanjing Medical University, Nanjing, China; 10 Department of Colorectal and Anal Surgery, The First Affiliated Hospital of Shandong First Medical University, Jinan, China; 11 Department of Colorectal Surgery, Union Hospital, Fujian Medical University, 29 Xin-Quan Road, Fuzhou, Fujian 350001 China

**Keywords:** Rectal neoplasms, Carcinoma, Neuroendocrine, Recurrence, Nomograms

## Abstract

**Background:**

The prognostic nutritional index (PNI), alkaline phosphatase (ALP), and lymph node ratio (LNR) are reportedly related to prognosis. The aim of this study was to elucidate the clinical importance of the LNR and hematological parameters in patients with high grade rectal neuroendocrine neoplasms (HG-RNENs) who were undergoing radical resection.

**Methods:**

We reviewed the medical records of patients with HG-RNENs from 17 large-scale medical centers in China (January 1, 2010–April 30, 2022). A nomogram was constructed by using a proportional hazard model. Bootstrap method was used to draw calibration plots to validate the reproducibility of the model. Concordance index (C-Index), decision curve analysis (DCA), and time-dependent area under the receiver operating characteristic curve (TD-AUC) analysis were used to compare the prognostic predictive power of the new model with American Joint Committee on Cancer (AJCC) TNM staging and European Neuroendocrine Tumor Society (ENETS) TNM staging.

**Results:**

A total of 85 patients with HG-RNENs were enrolled in this study. In the 45 patients with HG-RNENs who underwent radical resection, PNI ≤ 49.13 (HR: 3.997, 95% CI: 1.379–11.581, *P* = 0.011), ALP > 100.0 U/L (HR: 3.051, 95% CI: 1.011–9.205, *P* = 0.048), and LNR > 0.40 (HR: 6.639, 95% CI: 2.224–19.817, *P* = 0.0007) were independent predictors of relapse-free survival. The calibration plots suggested that the nomogram constructed based on the three aforementioned factors had good reproducibility. The novel nomogram revealed a C-index superior to AJCC TNM staging (0.782 vs 0.712) and ENETS TNM staging (0.782 vs 0.657). Also, the new model performed better compared to AJCC TNM staging and ENETS TNM staging in DCA and TD-AUC analyses.

**Conclusions:**

LNR, ALP, and PNI were independent prognostic factors in patients with HG-RNENs after radical resection, and the combined indicator had better predictive efficacy compared with AJCC TNM staging and ENETS TNM staging.

## Introduction

Neuroendocrine neoplasms (NENs) are rare neoplasms arising from peptidergic neurons and neuroendocrine cells; they can demonstrate neuroendocrine differentiation and express neuroendocrine markers [1]. These neoplasms are mostly found in the gastrointestinal tract, pancreas, and lung [2], with the rectum being the third most frequent site. The incidence of rectal neuroendocrine tumors has increased and was 1.3/100,000 from 2007 to 2016 [2, 3]. Rectal neuroendocrine neoplasms (R-NENs) are classified into rectal neuroendocrine tumor (R-NET), rectal neuroendocrine carcinoma (R-NEC), and rectal mixed adeno-neuroendocrine carcinoma (R-MANEC) based on differentiation degree, mitotic count, and Ki-67 index. Among these, rectal neuroendocrine carcinoma and mixed adeno-neuroendocrine carcinoma are also known as high grade rectal NENs (HG-RNENs), which have worse prognosis and are associated with higher metastatic risk than R-NET [4].

Surgical resection remains the preferred treatment option for primary localized HG-RNENs [4]. However, despite curative resection, the prognosis of HG-RNENs is still poor [5]. The American Joint Committee on Cancer (AJCC) and the European Association for Neuroendocrine Tumor (ENETS) TNM staging system are the most widely used prognostic evaluation method for HG-RNENs after radical resection. They divide HG-RNENs into stage I–IV based on T stage, number of positive lymph nodes (PLN), and distant metastasis [6, 7]. However, recent studies suggest that lymph node ratio (LNR) is a better prognostic predictor than PLN [8, 9]. Moreover, preoperative hematological parameters like prognostic nutritional index (PNI) and alkaline phosphatase (ALP) also demonstrate excellent prognostic efficacy [10–13]. Currently, simple and effective prognostic model containing preoperative hematological parameters and pathological parameters is still lacking for HG-RNENs. Therefore, it is essential to establish an accurate prognostic prediction model to individualize treatment in HG-RNENs patients.

In this study, we retrospectively collected data from 17 large medical centers in China to construct a prognostic prediction model for patients with HG-RNENs. Furthermore, we compared the model with the traditional AJCC TNM staging and ENETS TNM staging to determine their prognostic predictive value.

## Materials and methods

### Patients and data collection

**Fig. 1:**
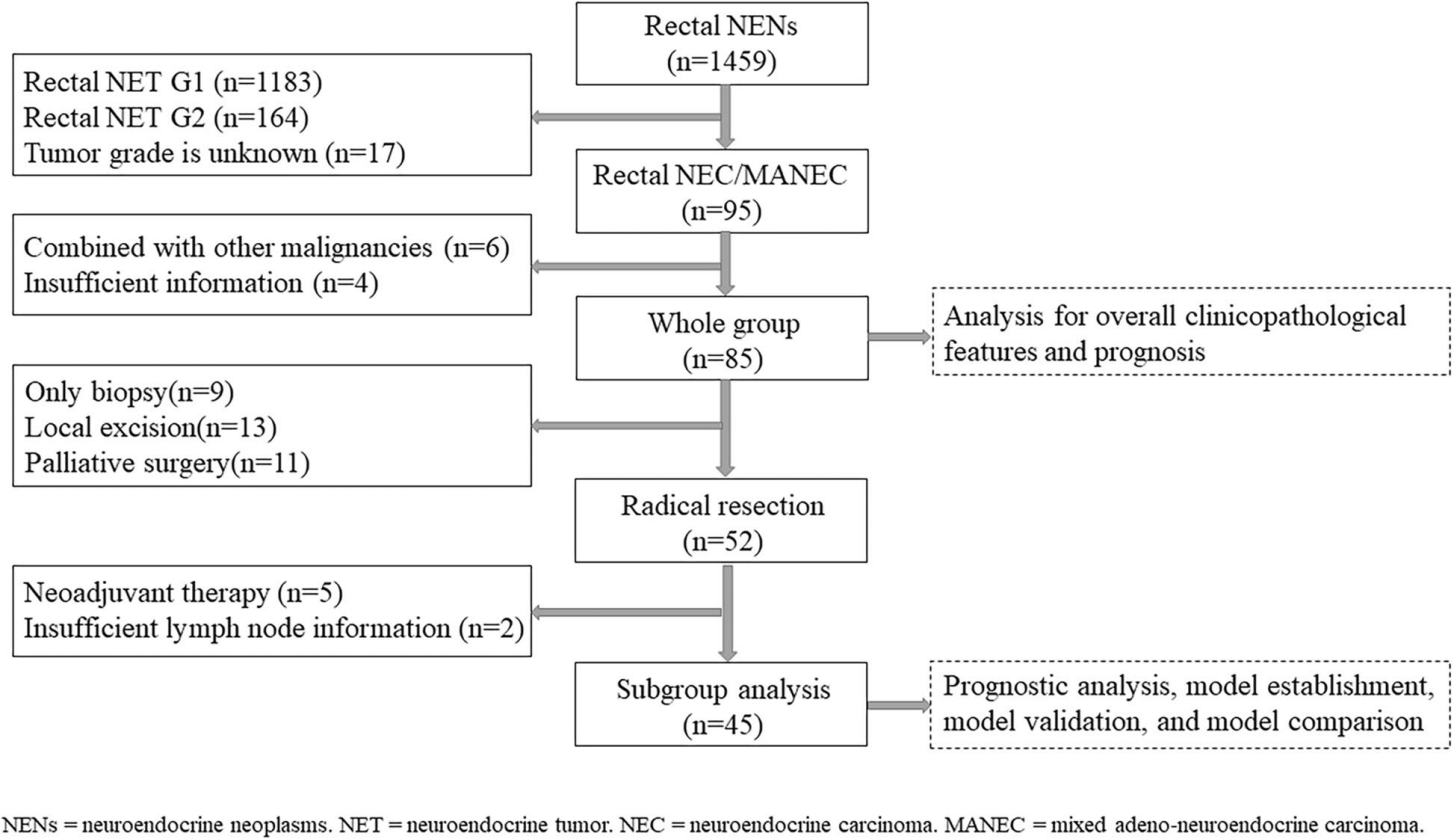
Patient selection flowchart

We conducted a retrospective study of HG-RNENs patients in 17 large-scale medical centers in China, spanning from January 1, 2010, to April 30, 2022. Demographic, clinicopathologic, treatment, and outcome data were extracted from the electronic medical records of each hospital by surveyors with expertise in NENs, using standardized data collection templates. The inclusion criteria were as follows: (1) confirmation of neuroendocrine neoplasms by pathology, (2) tumor grade of R-NEC or R-MANEC, and (3) primary rectal neuroendocrine neoplasms. The exclusion criteria were as follows: (1) comorbidity with other malignancies and (2) incomplete clinical data or follow-up information. A total of 85 out of 1459 patients met our study criteria. The procedure for extracting eligible study subjects is shown in Fig. 1.

### Criteria

We collected blood routine test records within 7 days before radical resection and calculated the PNI from serum albumin and peripheral lymphocyte counts. For patients who underwent radical resection with lymphadenectomy, we obtained lymph node–related information, with PLN defined as the number of positive lymph nodes and LNR defined as the ratio of the number of positive lymph nodes to the number of dissected lymph nodes.

Tumor size was determined based on the longest diameter of the tumor recorded in the pathology report. In cases where patients only underwent biopsy, tumor size was determined based on endoscopic findings before treatment. For patients who underwent palliative surgery, tumor size was assessed based on imaging reports. Tumor stage was classified according to the AJCC and ENETS TNM staging system, and tumor grade was classified based on the WHO 2010 classification. The mitosis count was expressed as the number of mitotic cells in ten high-power fields from hematoxylin and eosin stained slides examined with microscopy, and the Ki-67 index was calculated as the percentage of cells labeled by immunohistochemistry.

### Follow-up

We defined cancer-specific survival (CSS) as the interval from diagnosis to death attributed to neuroendocrine neoplasms and relapse-free survival (RFS) for patients in whom neoplasms were removed as the interval from the date of intervention to the date of recurrence. All patients were followed up through telephone, outpatient, and inpatient means. Patients after complete resection were followed every 3~6 months and annually after 5 years, while patients who underwent palliative resection or biopsy were followed every 3 months. We conducted the last follow-up in July 2022. Follow-up exams included routine blood tests, chromogranin A tests, chest CT scans, and whole abdominal and pelvic contrast-enhanced CT or MRI; PET-CT was performed if recurrence or metastasis was suspected. Loss to follow-up was defined as failure to contact either the patient or their family.

### Statistical analysis

We presented continuous variables as the median with interquartile range and categorical data as numbers and percentages. CSS and RFS were analyzed using the Kaplan-Meier method, and variables were compared using the log-rank test in univariable analysis and Cox proportional hazard regression in multivariable analysis.

We used the R software (version 4.0.0) for statistical analysis and the X-tile software to determine the optimal cut-off value of continuous variables. Variables with a *P* < 0.10 in the univariate analysis were included in the multivariate analysis, and *P* < 0.05 was considered statistically significant.

### Establishment and validation of nomogram

The independent prognostic factors identified by multivariate analysis are integrated to draw the nomogram by using the “rms” package of the R software (version 4.0.0). The average predicted survival rate was compared with the average actual survival rate, and calibration plot was used to estimate the precise predictions for 1-, 2-, and 3-year RFS. DCA analysis was performed to evaluate the clinical utility of the nomogram based on net benefits at different threshold probabilities. The time-dependent area under the receiver operating characteristic curve (TD-AUC) analysis was calculated and plotted by using the “timeROC” package of the R software (version 4.0.0).

## Results

### Demographic and clinicopathology characteristics

**Fig. 2:**
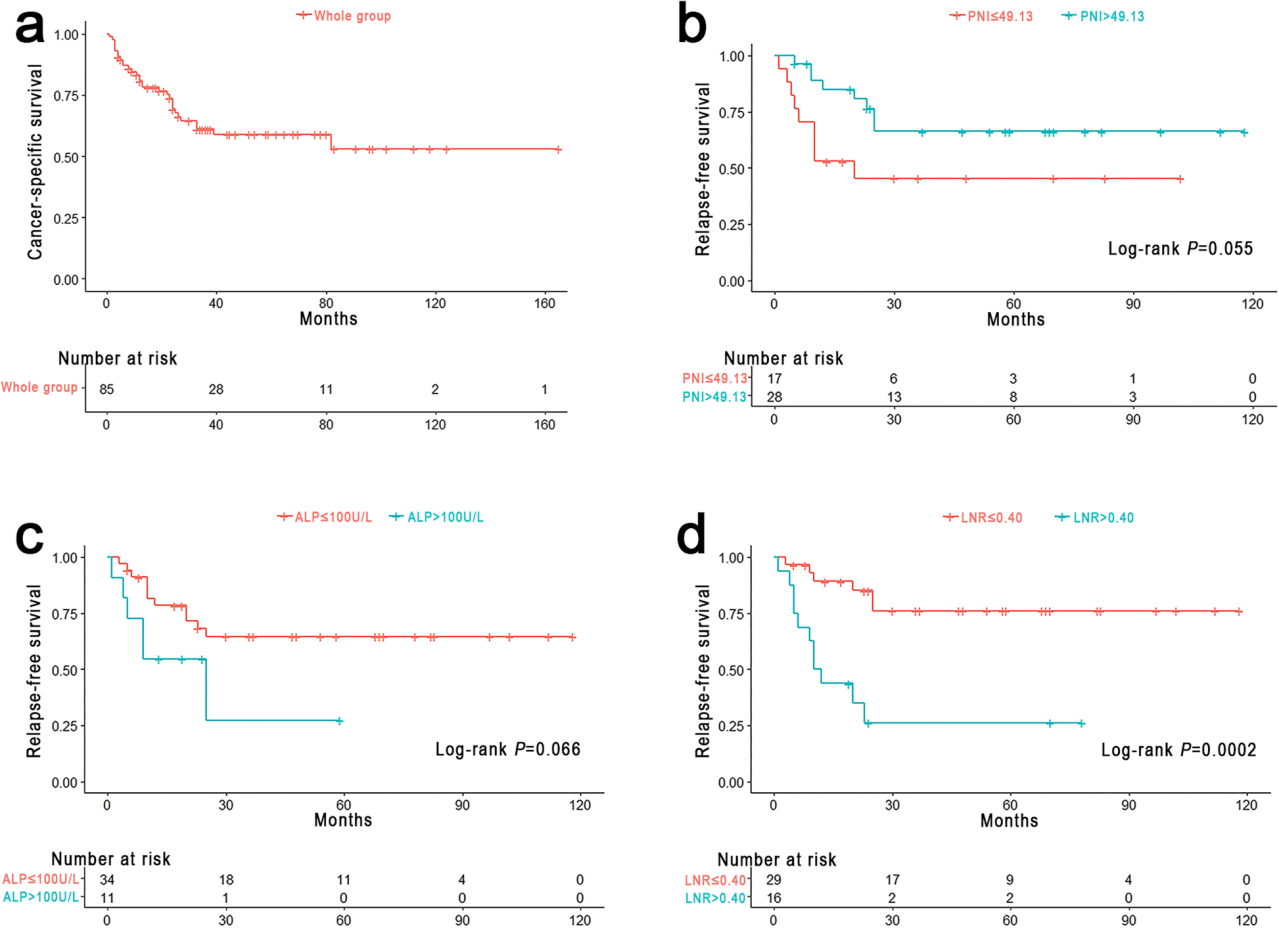
**a** Cancer specific survival for the whole group. **b** Relapse-free survival stratified by PNI. **c** Relapse-free survival stratified by ALP. **d** Relapse-free survival stratified by LNR

A total of 85 patients with HG-RNENs were included in this study, with 50 males (58.8%) and 35 females (41.2%), at a median age of 57.0 (52.0–66.0) years. The median tumor size was 3.0 (2.0–5.5) cm, and the distance from the anus was 6.0 (4.0–10.0) cm. Tumor staging revealed that 12 (14.1%) patients were staged as T1, 18 (21.2%) as T2, 36 (42.4%) as T3, and 19 (22.4%) as T4. Thirteen (15.3%) patients underwent local excision, 52 (61.2%) underwent radical resection, and 20 (23.5%) underwent palliative surgery or biopsy for distant metastasis. Of the 63 patients who underwent radical or palliative resection, 41 (65.1%) had clinically positive lymph nodes based on the histopathology results. Seven of 85 patients (8.2%) received neoadjuvant therapy and 41 (48.2%) received adjuvant therapy. See Table 1 for a summary of the patients’ demographic and clinicopathological features. The 1-year, 3-year, and 5-year CSS for the whole group of patients with HG-RNENS were 80.7%, 61.1%, and 59.0%, respectively (Fig. 2a).

**Table 1:** Clinicopathological data of patients with high grade rectal neuroendocrine neoplasms

Clinicopathological character	Total *n* = 85	
	*n*	IQR or %
Sex, *n* (%)		
Female	35	41.2%
Male	50	58.8%
Age, year (IQR^a^)	57.0	(52.0–66.0)
Tumor size, cm (IQR)	3.0	(2.0–5.5)
Distance from the anus, cm (IQR)	6.0	(4.0–10.0)
Ki-67, % (IQR)	60.0	(40.0–80.0)
T stage, *n* (%)		
T1	12	14.1%
T2	18	21.2%
T3	36	42.4%
T4	19	22.4%
Lymph node metastasis, *n* (%)^b^		
Negative	22	34.9%
Positive	41	65.1%
Evaluate lymph nodes, *n* (IQR)^b^	14.0	(6.0–17.0)
Positive lymph nodes, *n* (IQR)^b^	1.0	(0.0–5.0)
LNR, (IQR)^b^	0.07	(0.00–0.44)
Distant metastasis, *n* (%)		
Negative	65	76.5%
Positive	20	23.5%
Neoadjuvant therapy, *n* (%)		
No	78	91.8%
Yes	7	8.2%
Surgical type, *n* (%)		
Only biopsy	9	10.6%
Local excision	13	15.3%
Radical resection	52	61.2%
Palliative surgery	11	12.9%
Adjuvant therapy, *n* (%)		
No	44	51.8%
Platinum-based regimen	25	29.4%
Other chemotherapy	16	18.8%
Microvascular invasion, *n* (%)		
Negative	69	81.2%
Positive	16	18.8%
Perineural invasion, *n* (%)		
Negative	71	83.5%
Positive	14	16.5%

^a^*IQR* Interquartile range

^b^Only for patients who underwent rectal resection or palliative surgery (i.e., low anterior resection and abdominoperineal resection) with available lymph node-related information

### Prognostic analysis for HG-RNENS patients who underwent radical resection

As shown in Fig. 1, 45 patients who met the following criteria were included in the subsequent analysis of HG-RNENS patients who underwent radical resection: (1) no neoadjuvant therapy, (2) radical resection + lymphadenectomy, and (3) available lymph node information. The prognostic univariate analysis revealed that PNI (*P* = 0.055), ALP (*P* = 0.066), and LNR (*P* = 0.0002) were associated with RFS. Multivariate analysis showed that PNI ≤ 49.13 (HR: 3.997, 95% CI: 1.379–11.581, *P* = 0.011), ALP > 100.0 U/L (HR: 3.051, 95% CI: 1.011–9.205, *P* = 0.048), and LNR > 0.40 (HR: 6.639, 95% CI: 2.224–19.817, *P* = 0.0007) were independent predictors of RFS. The log-rank test and Cox proportional hazards regression analysis for RFS are shown in Table 2. Patients with PNI ≤ 49.13 had a 3-year RFS of 45.4%, whereas patients with PNI > 49.13 had a 3-year RFS of 66.2%. Patients with ALP ≤ 100.0 U/L had a 3-year RFS of 64.7%, whereas patients with ALP > 100.0 U/L had a 3-year RFS of 27.3%. Patients with LNR ≤ 0.40 had a 3-year RFS of 76.1%, whereas patients with LNR > 0.40 had a 3-year RFS of 26.3%. The survival curves for PNI, ALP, and LNR in different groups are shown in Fig. 2b–d.

**Table 2:** Univariable and multivariable analysis of factors associated with relapse-free survival in patients with high grade rectal neuroendocrine neoplasms

Clinicopathological factors	Univariate analysis				Multivariable analysis	
	HR^a^	95% CI^b^	*P* value	HR	95% CI	*P* value
Sex (female/male)	0.964	0.372–2.495	0.938			
Age (> 65/ ≤ 65)	2.133	0.590–7.714	0.138			
Tumor size (> 2.0 cm/ ≤ 2.0 cm)	0.497	0.141–1.755	0.174			
PLR (> 116.0/ ≤ 116.0)	1.719	0.656–4.508	0.295			
NLR (> 2.45/ ≤ 2.45)	1.815	0.660–4.992	0.205			
PNI (≤ 49.13/ > 49.13)	**2.431**	**0.875**–**6.755**	**0.055**	**3.997**	**1.379**–**11.581**	**0.011**
ALP (> 100.0 U/L/ ≤ 100.0 U/L)	**2.428**	**0.711**–**8.290**	**0.066**	**3.051**	**1.011**–**9.205**	**0.048**
Ki-67 (> 65%/ ≤ 65%)	1.232	0.475–3.197	0.667			
Histology (NEC/MANEC)	0.563	0.167–1.890	0.267			
T stage (T3 + T4/T1 + T2)	1.731	0.594–5.047	0.376			
LNR (> 0.40/ ≤ 0.40)	**5.183**	**1.757**–**15.290**	**0.0002**	**6.639**	**2.224**–**19.817**	**0.0007**
Microvascular invasion (positive/negative)	1.058	0.297–3.770	0.928			
Perineural invasion (positive/negative)	0.743	0.198–2.793	0.688			

Analysis for patients who underwent radical resection + lymphadenectomy with available lymph node information and did not receive neoadjuvant therapy

^a^*HR* Hazard ratio

^b^*CI* Confidence interval

### Establishment and validation of nomogram

**Fig. 3:**
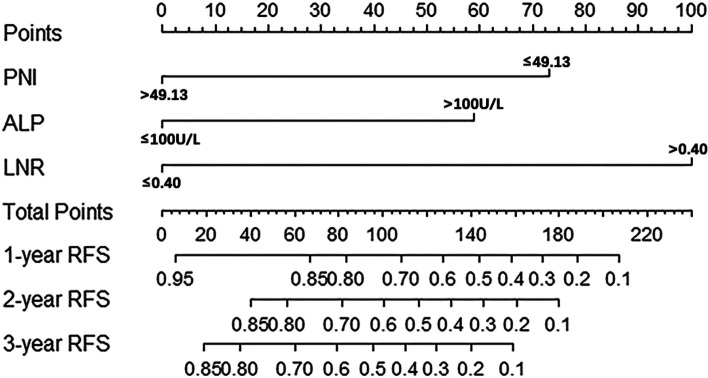
Nomogram

**Fig. 4:**
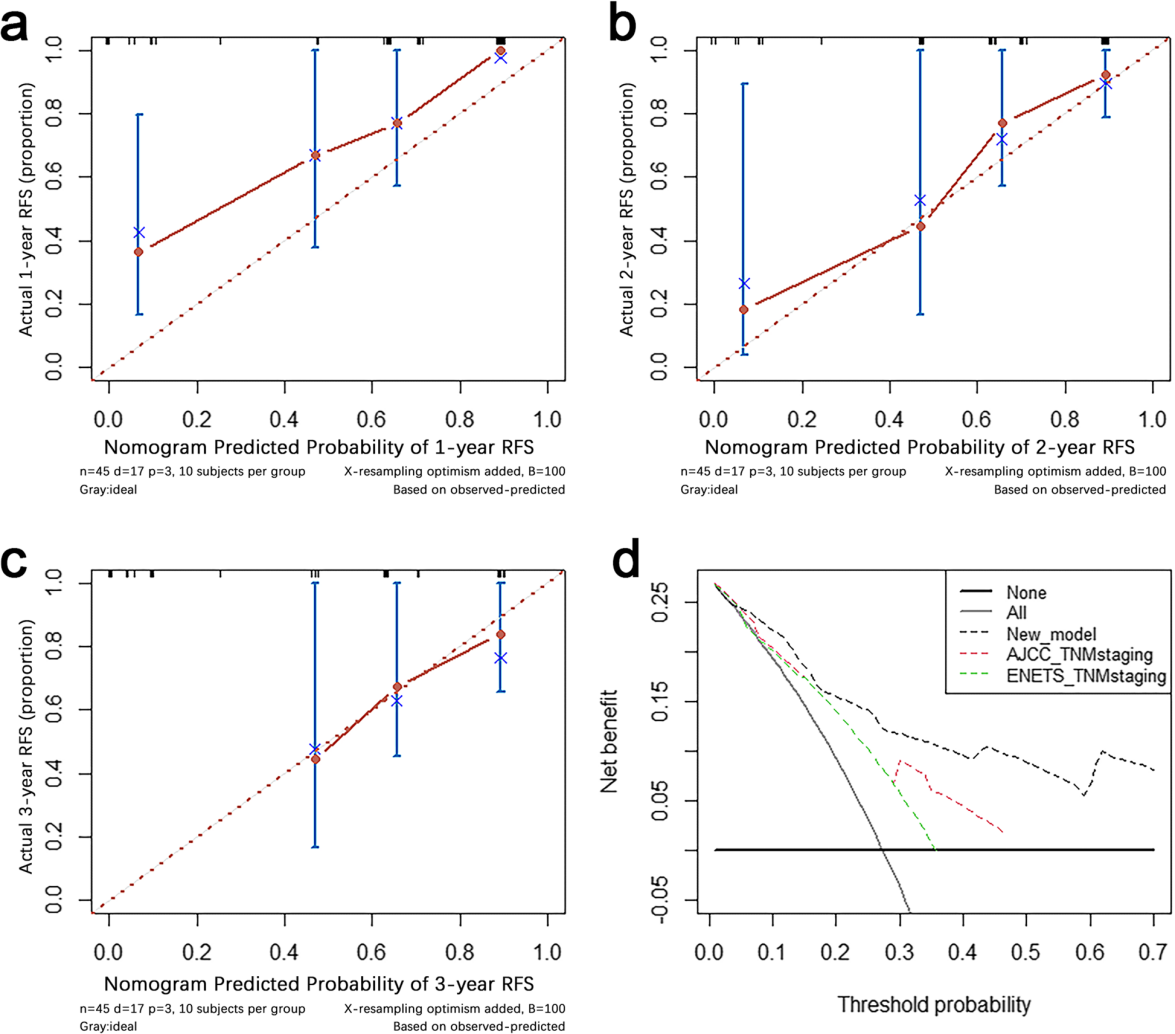
**a** Calibration plot of 1-year relapse-free survival. **b** Calibration plot of 2-year relapse-free survival. **c** Calibration plot of 3-year relapse-free survival. **d** The decision curve analysis (DCA)

Based on the three independent predictors, we established a nomogram for the RFS of HG-RNENS patients. LNR had the largest weight in the nomogram, followed by PNI and ALP, as shown in Fig. 3. The accuracy of nomograms is verified by using bootstrap resample. The similarity between the actual survival rate and the predicted survival rate of nomograms was verified by the calibration plot (Fig. 4a–c). The actual survival rate and the survival rate predicted by nomogram were on the *X*-axis and *Y*-axis, respectively, using the Kaplan-Meier method. The calibration plot indicated that the nomogram predicted the 1-year, 2-year, and 3-year RFS rates of the patients with HG-RNENS.

### Comparison of nomogram and TNM staging

**Fig. 5:**
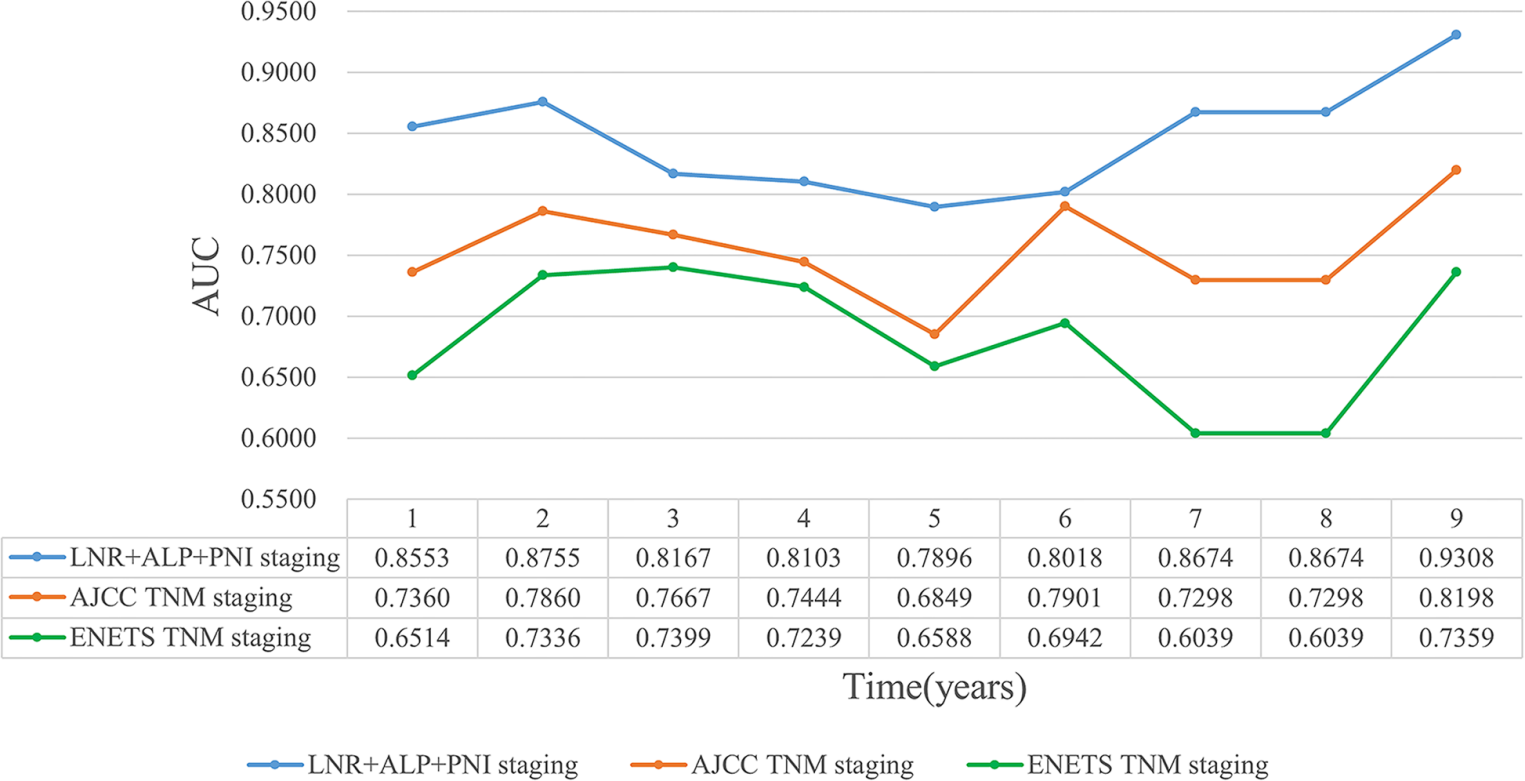
Time-dependent area under the receiver operating characteristic curve (TD-AUC) analysis for relapse-free survival

The C-index of our novel nomogram was higher than that of ENETS TNM staging (0.782 vs 0.657) and AJCC TNM staging (0.782 vs 0.712) in predicting RFS. Predicted net benefit was compared between the new model and TNM staging using DCA analysis. The DCA curve indicated that the nomogram was feasible for making valuable and informed judgments of the prognosis. Our analysis suggested that if the threshold probability of recurrence in patients was approximately 0–70% predicted by this nomogram, the use of this nomogram to guide treatment measures in patients with HG-RNENS would provide more benefit than either the “treat all patients” or the “treat none patient” schemes. Also, the DCA decision curve suggests that the net yield of the new model is higher than that of AJCC TNM staging and ENETS TNM staging in the range of threshold 10–40% (Fig. 4d). Furthermore, the time-dependent ROC curve of the new model was found to be consistently more favorable in predicting RFS from one to nine years, as shown in Fig. 5.

## Discussion

HG-RNENS is a highly heterogeneous group of neoplasms that can present either as low-grade malignant or as highly aggressive [14]. The incidence of HG-RNENS has increased in recent years, mainly due to increased health awareness and advances in endoscopic techniques [15]. Most HG-RNENS are nonfunctional neoplasms, which means that these patients have no symptoms related to hormone secretion and lack specific clinical manifestations; thus, HG-RNENS is mostly diagnosed at the time of advanced stage [16]. In this study, more than half of the patients developed lymph node metastasis at the time of diagnosis, and about a quarter developed distant metastasis, with a 5-year CSS of only 59.0% in the whole group. This suggests that the overall prognosis of HG-RNENS is poor. Thus, in addition to conventional contrast-enhanced imaging, clinicians should perform EUS, PET-CT, pathological biopsy, and other examinations to accurately assess tumor pathological grade, invasion condition, lymph node, and distant metastasis, which may be essential for choosing the most appropriate treatment. In the further analysis, we constructed a prognostic predictive model based on clinicopathologic parameters that significantly impact the prognosis of patients with HG-RNENS. To our knowledge, this is the first prognostic model study for HG-RNENS.

Recent studies have shown that some preoperative hematological parameters are helpful to predict the prognosis of patients with digestive tract tumors. Tokunaga et al. retrospectively analyzed 468 patients with colorectal cancer who underwent radical resection, and the results showed that using 45.0 as the cutoff value of PNI can predict OS and RFS effectively [10]. Also, Tominaga et al. suggested that distinguishing PNI by 42.4 could effectively predict the prognosis of elderly patients with colorectal cancer [11]. These studies suggested that PNI could be used as an effective predictor of the prognosis of patients with colorectal cancer, and low PNI was often associated with adverse prognostic events. Hu et al. included 61 patients with gastric cancer, and the results showed that the prognosis of patients with ALP ≤ 225.0 U/L was significantly better than that of patients with ALP > 225.0 U/L [12]. Chau I et al. also obtained similar conclusions in the study of esophagogastric junction cancer; their study showed that using 100.0 U/L as the cutoff value of ALP could effectively predict the prognosis of patients with esophagogastric junction cancer [13]. In the analysis of this study, PNI ≤ 49.13 and ALP > 100 U/L were both effective prognostic predictors in patients with HG-RNENs.

Lymph node metastasis is a risk factor for the prognosis of RNENs patients, and PLN-based TNM staging system is currently the most widely used staging system [6, 7]. The PLN staging system depends only on the number of positive nodes and does not consider the influence of the number of dissected nodes/numbers of negative nodes. Studies have found that even in patients with the same number of positive lymph nodes, insufficient number of dissected lymph nodes might indicate a poor prognosis [8]. In contrast to PLN, LNR is less susceptible to the number of dissected lymph nodes during surgery and is thus more stable. As a novel pathological factor, LNR has been demonstrated to be associated with the prognosis of diseases such as gastric cancer, colorectal cancer, and esophageal squamous cell carcinoma [17–19]. In this study, we found that LNR > 0.40 was associated with adverse prognostic events for HG-RNENS. Our new model based on LNR, ALP, and PNI showed better predictive power than AJCC TNM staging and ENETS TNM staging in C-index and DCA and TD-AUC analyses and demonstrated better predictive power.

Predictive models, including scoring model and nomogram, have been widely studied and applied in the clinical practice of NENs, but such models are still lacking for the diagnosis and treatment of HG-RNENS [20–22]. In this study, we constructed an accurate and effective prognostic prediction model for RFS in HG-RNENS patients based on the three aforementioned independent predictors. In the establishment of RFS prognostic model, the novel nomogram showed good predictive efficacy with C-index of 0.782. This suggests that the new model based on LNR, ALP, and PNI constructed in this study has a high predictive value for the prognosis of HG-RNENS patients. Given its simplicity and effectiveness, the new model can help clinicians select treatment modalities and even help screen patients suitable for clinical research.

This study has some limitations. First, although we collected multicenter data to reduce potential bias, the nature of the retrospective study still impacts the statistical power of this study and reduces clinical value. Second, limited by the incidence of HG-RNENS, the effectiveness of the predictive model was studied only through a small sample. Although we have verified and compared the models, the clinical effect of this model still needs to be further verified by international multicenter studies with larger sample size. However, to our knowledge, this is currently the study with the largest sample size for HG-RNENS and can provide some guidance for the diagnosis and treatment selection of HG-RNENS.

## Conclusions

In summary, LNR, ALP, and PNI were found to be independent predictors of prognosis in HG-RNENS in this study. In addition, we constructed a predictive model for RFS in patients with HG-RNENS after radical resection based on data from multicenter data, which showed better predictive power than AJCC TNM staging and ENETS TNM staging. The new model can effectively predict RFS and can be used to guide the development of treatment options for patients with HG-RNENS.

## Data Availability

The datasets used and analyzed during the current study are available from the corresponding author on reasonable request.

## Abbreviations

PNI: Prognostic nutritional index
ALP: Alkaline phosphatase
LNR: Lymph node ratio
HG-RNENs: High grade rectal neuroendocrine neoplasms
C-Index: Concordance index
DCA: Decision curve analysis
TD-AUC: Time-dependent area under the receiver operating characteristic curve
AJCC: American Joint Committee on Cancer
ENETS: European Neuroendocrine Tumor Society
NENs: Neuroendocrine neoplasms
R-NENs: Rectal neuroendocrine neoplasms
R-NET: Rectal neuroendocrine tumor
R-NEC: Rectal neuroendocrine carcinoma
R-MANEC: Rectal mixed adeno-neuroendocrine carcinoma
PLN: Positive lymph nodes
CSS: Cancer-specific survival
RFS: Relapse-free survival
